# Mass transfer intensification for carbon quantum dot nanofluid drops under pulsed electric fields

**DOI:** 10.1038/s41598-022-16663-9

**Published:** 2022-07-16

**Authors:** Farnaz Jafari, Javad Saien, Alimorad Rashidi

**Affiliations:** 1 Bu-Ali Sina University, Hamedan, Iran; 2 Carbon and Nanotechnology Research Center, RIPI, Tehran, Iran

**Keywords:** Quantum dots, Fluidics, Chemical engineering

## Abstract

Simultaneous use of carbon quantum dot (CQD) nanofluids and pulsed electric fields exhibits amazing mass transfer intensification in liquid–liquid extraction of circulating drops. Here, the chemical system of kerosene–acetic acid–water with mass transfer resistance in the organic phase was used in which organic nanofluid drops contained CQD or modified CQD-Fe. These products with extremely small sizes of 7.2 and 13.4 nm were synthesized and characterized by DLS, Zeta potential, XRD, EDS and SEM techniques. To find optimum conditions, CQD concentrations within (0.0005–0.003) wt%, electric field frequencies within (50–550) Hz and electric field strengths to 16 V/cm were examined. From hydrodynamic point of view, the flow pattern of drops was in circulating mode, and that terminal velocity of drops correctly followed the Grace model. The substantial effect of pulsed electric field on the CQD and CQD-Fe nanofluids, brought about mass transfer enhancements to 263.5 and 291.6%. This can be attributed to the electro-induced motion of global CQDs with pulsed electric fields. For the aim of modelling, the adapted Kumar and Hartland equation with a developed correlation of the enhancement factor versus involved dimensionless variables were satisfactory to reproduce the mass transfer coefficient data.

## Introduction

Mass transfer intensification in liquid–liquid extraction has the significance of improving efficiency and reducing the energy consumption. Several mass transfer intensification approaches have been introduced as^1^:

- Changing hydrodynamic of drops from rigid to circulating or oscillating.
- Improving interfacial instabilities to assist momentum and mass transfer across the interface.
- Altering operating conditions such as pH, temperature, and applying ultrasound.
- Using additives like salts and nanoparticles.

A preferred approach for mass transfer intensification is the use of nanofluids. Nanofluids, defined as stable and uniform dispersion of nanoparticles in base fluids, are the promising generation of fluids with outstanding properties. Having the significant potential of various nanofluids in heat transfer, their applications in mass transfer enhancement has also attracted the attention of investigators since early 2011^2^.

Studies indicate that conventional fluids could be substituted with nanofluids for the aim of process intensification. A detailed review on utilizing nanofluids in various columns with different chemical systems has been presented by Amani et al.^3^ The main liable mechanism of the enhancing effect of nanofluids is related to the Brownian motion, random movement of nanoparticles, and subsequent internal convection. Small nanoparticles with lower density are more capable for this aim^1^. Accordingly, the influence of nanoparticle size has been separately studied in an irregular packed column and single drop extractor by utilizing the SiO_2_-toluene nanofluid^4,5^. The results have demonstrated that using small nanoparticles significantly improve the convection. Notably, in all the performed investigations, nanofluids have been of metal/metal oxide nanoparticles with rather large size, high density and probable toxic effects on the environment.

Carbon Quantum Dots (CQDs), with unique features of simple synthesis, stable chemical inertness, efficient light harvesting and photo-induced electron transfer provide beneficial potential for different applications^6,7^. Recently, the desired properties of CQD has been evolved through nanohybrids, which are the combination of two or more nanoscale materials usually with multiple functionality^8^. Considering easily functionalization of CQDs via reducing metal salts, valuable CQD-metal products have been achieved often with synergistic properties. In this regard, Azizi et al.^9^, for instance, investigated the synthesis and utilization of highly stable CQD-Cu nanofluid for heat transfer enhancement. Despite these and desired physicochemical properties such as low density, extremely small particle size and the possibility of surface functionalization, their application in mass transfer has not been investigated yet.

Applying external fields as an innovative approach for mass transfer intensification has also been confirmed in several investigations. In this regard, ultrasonic external field has been employed for the cumene–isobutyric acid–water chemical system^10^. Growth of mass transfer with ultrasound irradiation has been attributed mainly to the intensified motion of nanoparticles and consequent internal convection. In a relevant study, the positive effect of oscillating magnetic field (0.36–1.45 T, constant frequency of 41 Hz) on the magnetite-toluene nanofluid has been reported^11^. It was demonstrated that the motion of magnetite nanoparticles, under two established directions of magnetic field, was significantly increased and in turn, promoted the mass transfer coefficient. In 2016, an investigation on the capability of magnetic field with controllable strength and frequency on mass transfer of single drops revealed that increasing field strength led to nanoparticle motions and promoting micro convections^12^. An optimum magnetic field frequency relevant to the highest mass transfer rate was found. The remarkable point is that application of ultrasonic field in extraction column could not be widely developed due to high energy consumption, and that effect of magnetic field depends on the magnetic properties of nanoparticles. Therefore, limited kinds of nanoparticles are influenced by magnetic fields.

To overcome these drawbacks, electric field as a cost-effective, simple design and ease of control seems consistent. Utilizing electric field on nanofluids, can remarkably intensify the mass transfer due to the fact that the movement of nanoparticles provides controllable electro-induced motion in addition to the random Brownian motion^13^. Indeed, the electric double layer around the nanoparticles and Zeta potential on the surface of conductive nanoparticles can be influenced by electric field through electro-induced forces which in turn, boost internal convection and mass transfer efficiency^14^.

The nanoparticle motion can be highly evolved by external bipolar pulsed electric fields with continuous displacement of positive and negative poles. Difference between dielectric constant of nanoparticles and the basefluid provides the electric gradient which could induce chaotic advection and turbulence^15^. Therefore, small nanoparticles with high electric charge density is preferred for utilizing under a pulsed electric field. Positively, the electrical conductivity of nanofluids is related to the nature of nanoparticles^16^. Thus, modification of nanoparticles with the aim of improving electrical properties (Zeta potential and electrical conductivity) is beneficial.

**Figure 1:**
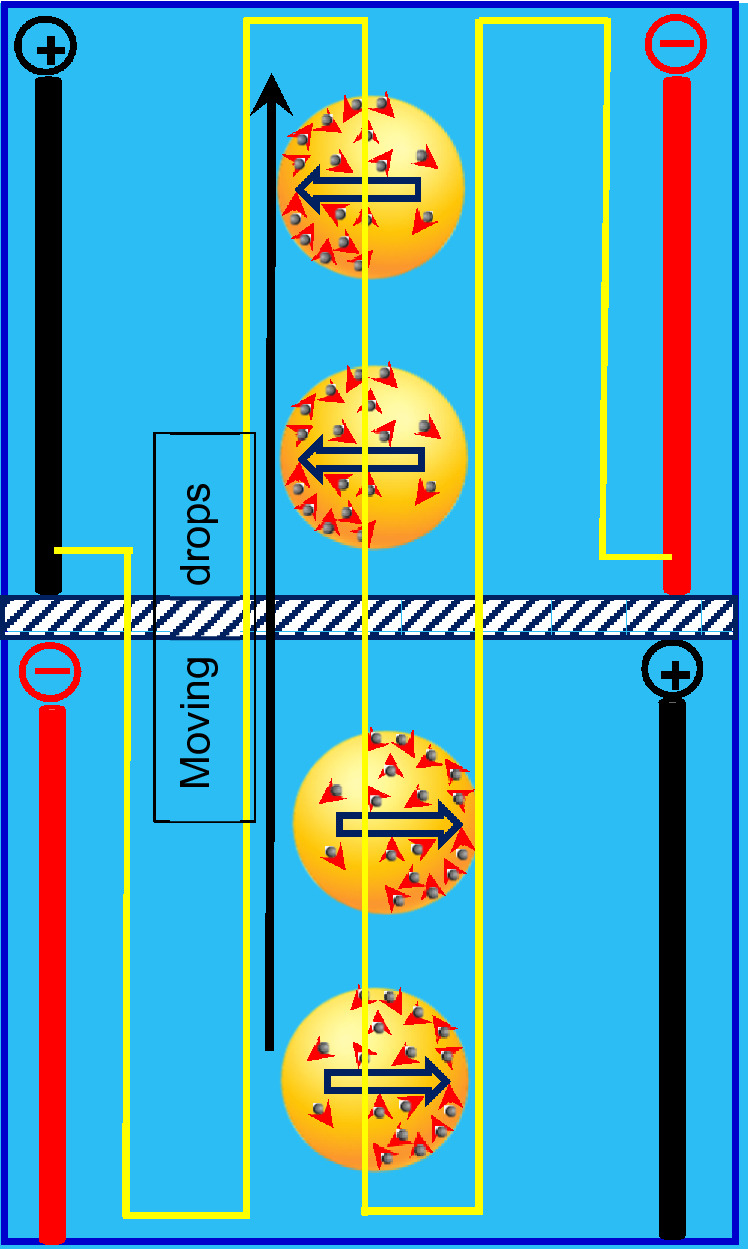
Schematic of nanofluid drops with effect of a pulsed electric field; Brownian and electro-induced motions are assigned with short and long arrows.

Despite so many substantial impacts, explored in the field of heat transfer, the benefits of applying electric field in mass transfer of nanofluids has not been investigated yet. Mass transfer intensification in liquid–liquid extraction is a major field for investigating this matter. The schematic of nanoparticles movements inside moving drops of an extraction process, under a pulsed electric field, is illustrated in Fig. 1 where Brownian and electro-induced motions are assigned with short and long arrows. Worth mentioning, despite the fact that electro-induced motion of nanoparticles is slower than Brownian motion, nanoparticles can travel longer distances owing to switching positive and negative bipolar electrodes^17^.

The present work aims to scrutinize the simultaneous use of CQD nanofluids and pulsed electric fields as a new approach for mass transfer intensification in drop dispersion operations. Pulsed electric field can presumably boost the CQD motions due to high surface electric charge, low density and the very small particle size. Further, introducing CQD-Fe as a modified quantum dot with high electrical conductivity can establish efficient mass transfer under pulsed electric fields. Investigations are made on the hydrodynamic and mass transfer behavior of drops in the chemical system of kerosene–acetic acid–water with mass transfer resistance mainly in the organic phase. The correlation of mass transfer coefficients is also attempted.

## Experimental

### Materials

In the used chemical system of kerosene–acetic acid–water, kerosene (reagent grade) and acetic acid (> 99% purity), as the dispersed phase and the solute, were purchased form Sigma Aldrich and Merck, respectively. The continuous phase was fresh pure water. The concentration of acetic acid in collected samples was determined through titration method using 0.1 N sodium hydroxide (Merck product). CQDs were synthesized using citric acid (C_6_H_8_O_7_, > 99% purity), Ferrous chloride (FeCl_2_.4H_2_O, > 99% purity), Ferric chloride (FeCl_3_.6H_2_O, > 99% purity) and aqueous ammonia solution (NH_4_OH, 30 wt%) all were purchased from Merck. The solvent for preparing the nanoparticle was high quality deionized water (electrical conductivity of 0.08 mS cm^–1^).

### Synthesis and characterization of CQDs

CQDs were prepared by a facile hydrothermal method, as properly been described in our previous report^18^. Briefly, 2 g citric acid as the carbon source was dissolved in 20 mL of deionized water and vigorously stirred to achieve a homogenous solution. After transferring the mixture into a Teflon autoclave, temperature was raised to 200 °C for 8 h and the obtained product was dried and cooled to the ambient temperature.

A part of CQD product was modified with Fe functionalizing to obtain a higher electrical conductivity and Zeta potential. In this regard, after dissolving 99.5 mg of FeCl_2_.4H_2_O and 270 mg of FeCl_3_.6H_2_O in 30 mL deionized water, the mixture was stirred for 5 min. Then, 30 mL of the as-prepared CQD (1 mg/mL) was added to the mixture. By gradual addition of 2 mL ammonia solution while vigorous agitation, the color change from light brown to dark brown and finally to black were observed. Subsequently, it was heated up to 80 °C and stirred for 1 h at this temperature. After cooling the mixture, the resulting precipitate was washed with deionized water several times to reach pH 7. Then the product was separated from water using a magnet and dried at 60 °C overnight^18^.

The characterization of as-prepared products was performed using different methods and devices. Dynamic light scattering method (DLS, Horiba, SZ100) to determine size distribution in the base fluid, Zeta potential analysis (Horiba, SZ100) to quantify effective electrical surface charge, X-Ray diffraction (XRD, PHILIPS, PW1730) to determine the crystal structure and size of nanoparticles along with confirming the absence of impurities, and Fourier transform infra-red (FT-IR, ABB BOMEM, MB100) spectroscopy to identify the functional groups of CQD and CQD-Fe nanoparticles. The elemental composition of products was identified with energy dispersive X-ray spectroscopy (EDX, TESCAN, MIRA III, SAMX Detector). Finally, field emission scanning electron microscopy (FE-SEM, TESCAN, MIRA III) was used to demonstrate the morphology and size distribution of nanoparticles.

### Nanofluid preparation and physical properties

The CQD-based nanofluids were prepared by dispersing various concentrations of CQD and CQD-Fe within (0.0005–0.0030) wt% in a 2.5 wt% acetic acid solution in kerosene. The obtained suspension was sonicated using ultrasonic probe (SONOPLUS-HF 320, Germany) for three 10 min courses giving a complete distribution of suspensions. The product was utilized to generate drops via nozzles for extracting acetic acid.

**Figure 2:**
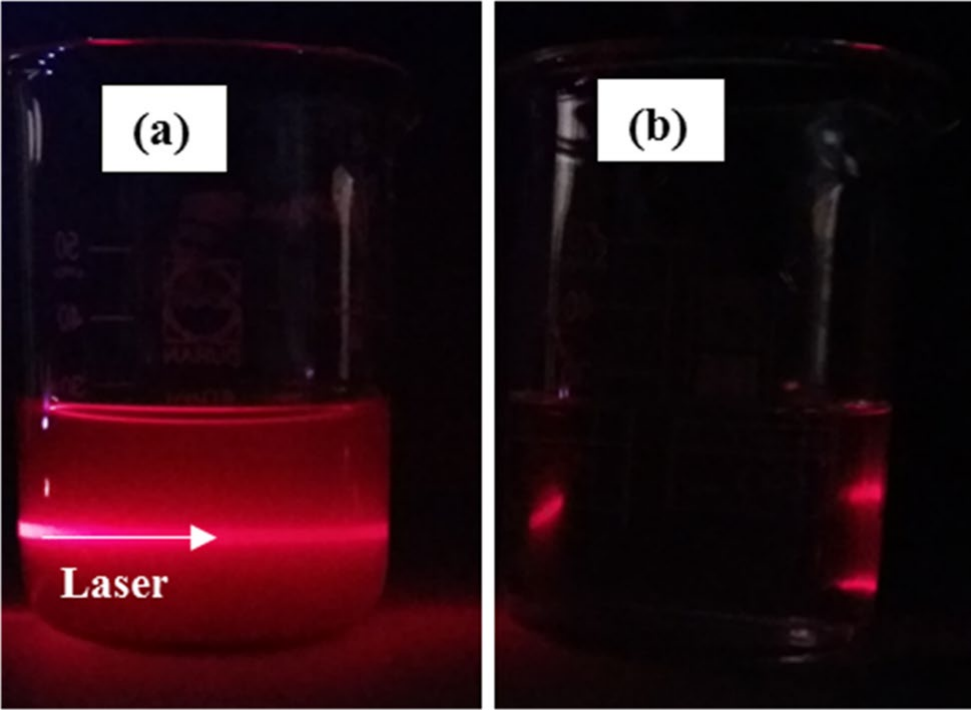
Tyndall effect for the CQD nanofluid (**a**) and the base fluid (**b**).

The Tyndall effect is corresponding to the light scattering by dispersed particles in suspensions, confirming the uniform dispersity of nanoparticles. As presented in Fig. 2, the observed direct laser light passing through the nanofluid confirms the well-dispersion of CQD nanoparticles.

**Figure 3:**
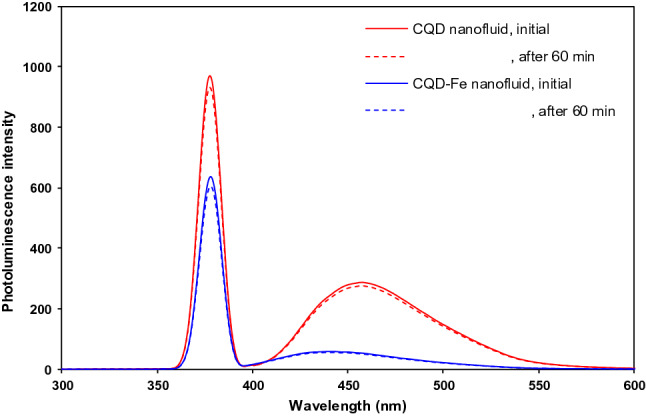
Photoluminescence spectra of CQD and CQD-Fe nanofluids just after preparation and after 60 min.

Further, for the aim of ensuring stability of dispersions, with respect to the linear relationship between the photoluminescence intensity and nanoparticle concentration, relevant spectra at different times can be considered^19^. A luminescence spectrometer (Perkin Elmer, LS 50 B, USA) was used in this regard. Figure 3 shows the CQD and CQD-Fe spectra for the maximum used nanoparticle concentration of 0.003 wt% samples under 360 nm excitation wavelength. As is obvious, confirming the nanofluid stability, there was no significant change in the intensity and emission wavelength for the initial nanofluid and after 60 min in rest. Thus, the stability of nanofluids remained satisfactorily over 95%, taking into account that the run times in this work did not exceed 25 min after each nanofluid preparation.

Table 1 presents the physical properties of the continuous and dispersed phases at 20 °C. The viscosity of each phase was measured with an Ubbleohde viscometer (uncertainty of 2 × 10^–3^ mPa·s) and based on Poiseville’s law^20^, $\mu = \rho (kt - {c \mathord{\left/ {\vphantom {c t}} \right. \kern-\nulldelimiterspace} t})$, where $\mu$, $\rho$ and $t$, respectively, indicate viscosity, density and efflux time in the viscometer capillary, and $k$ and $c$ stand for viscometer constants. The interfacial tension of phases was determined using the drop weight method as previously reported^21^. Dependency of the density of nanofluids on the concentration and the density of nanoparticles is as:

1 $$\rho_{nf} = (1 - \varphi )\rho_{bf} + \varphi \rho_{p}$$

where $\varphi$, $\rho_{p}$, $\rho_{nf}$, $\rho_{bf}$ stand for the particles volume fraction, and densities of particles (1291.4 and 1536.7 kg/m^3^ for CQD and CQD-Fe), nanofluid and the base fluid (kerosene, 792.37 kg/m^3^ at 20 °C), respectively. The volume fraction can be calculated from

2 $$\varphi = \left[ {\frac{{\omega_{p} \rho_{bf} }}{{(\omega_{p} \rho_{bf} ) + \rho_{p} (1 - \omega_{p} )}}} \right]$$

where $\omega_{p}$ is the particles weight fraction. The standard ASTM D4052 method was used to determine the density of CQDs^22^.

**Table 1:** The physical properties of the continuous and dispersed phases of the chemical system at 20 °C.

Phase	$\rho$(kg/m^3^)	$\mu$(mPa·s)	$D \times 10^{9}$(m^2^/s)	$\gamma$(mN/m)
Continuous (aqueous)	998.23	1.002	1.173	47.2
Dispersed:				
Base fluid (kerosene + 2.5 wt% acetic acid)	792.37	1.325	1.787	
CQD nanofluid (0.0005–0.003 wt%)	793.90–807.61	1.329–1.341		
CQD-Fe nanofluid (0.0005–0.003 wt%)	794.29–812.03	1.334–1.346		

For determinig the acetic acid molecular diffusivity in the dispersed and continuous phases, the well-known Wilke and Chang correlation was employed^23^. Consequently, the molecular weight of kerosene containing a complex mixture of hydrocarbons (C_10_–C_14_) should be essentially determined. The ASTM D86 distillation data for kerosene^24^ as well as the slope of the distillation curve give the mean average boiling point of 217.4 °C. The molecular weight of the kerosene was then attained as 182.2 g·mole by considering the density and the obtained mean average boiling point. Comprehensive relevant approach has been reported by Normand and Treil^25^.

### Procedure

The single drop extraction set-up consisted of a Pyrex glass column (internal diameter: 10 cm; height: 51 cm) and a syringe pump (JMS- SP 500, Japan) for generating different drop sizes via glass nozzles, placed at the bottom of the column. Symmetrical installation of two parallel stainless steel electrode with 5 cm distance inside the extraction column could establish a uniform bipolar pulsed electric field. Relevantly, an electric field generator with the capability of generating fine square pulses with variable electric field strengths ($E = {V \mathord{\left/ {\vphantom {V l}} \right. \kern-\nulldelimiterspace} l}$, $l$ is the distance between electrodes) and variable frequency was designed. In this regard, a function generator and an amplifier were used to adjust a certain frequency and a voltage, respectively. During each experiment, an oscilloscope (TRIO CS-1560A II, Japan) monitored the frequency and waveform constantly.

The dispersed phase of kerosene containing acetic acid and various concentration of CQD or CQD-Fe was flowed into the column, using the syringe and the connection tube to the nozzle within adjusted flow rate range of 40–130 mL/h. While passing drops through the water continuous phase in the column, the pulsed electric field was turned on and the initial (at a position 6 cm above the tip of nozzles) and final solute concentration (at the continuous phase level in the column) as well as the contact time and drop size were measured for each experiment. The size of drops was further determined through photographic and volumetric approaches. A high resolution camera (Cannon A590) took photographs from drops during the drops upward moving. Processing the images by an image processor software (Image J V.1.8), the precise size of drops was determined. It is worth mentioning that the possible interaction between two subsequent drops was prevented through adjusting dispersed phase flow rate. The contact time between initial and final points was measured and the corresponding terminal velocity values were easily obtained knowing the distance between these points (33 cm).

Upon reaching drops to the top level of the continuous phase, an inverted glass funnel attached to a vacuum bulb pipette collected them. Immediate pulling out the collected drops into the pipette gave a minimum contact area between phases at the funnel neck. The final concentration of solute in the collected samples was then analyzed by means of titration method using the standard 0.1 N sodium hydroxide solution and phenolphthalein as titrant and indicator, respectively. Noteworthy, that pH-metery was also utilized to confirm the endpoint of titration and excellent agreement was relevant. The operating temperature of experiments was at 20 ± 2 °C. The experimental runs were also conducted in another small column with the same nozzles, to determine the initial concentrations under steady movement of drops i.e. 6 cm above the tip of nozzles. The details of designed set-up have been previously reported^26^. Each experimental run was repeated at least three times and maximum deviation from average measured concentration values was less than 0.3%.

## Results and discussion

### Characterizations of the CQDs

**Figure 4:**
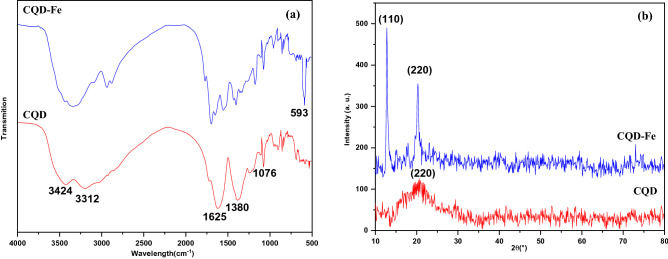
FT-IR spectrum (**a**) and XRD pattern (**b**) of the CQD and CQD-Fe products.

The FT-IR spectrum of the synthesized CQDs indicate which functional groups were present. Figure 4a shows the FT-IR spectrum for CQD and CQD-Fe, the observed peak at 3424 cm^–1^ corresponds to the stretching vibrations of hydroxyl groups; meanwhile, this broad peak joins up with the C–H stretching in the range of 2850–3300 cm^−1^ it can be attributed to the carboxylic acid group. Peaks at 1625 and 1076 cm^–1^ could be attributed to the stretching of carbonyl and stretching vibration of epoxides, respectively. The C–O stretching vibrations is indicated with the absorption peaks between wavenumber of 1000–1400 cm^−1^. Comparing FT-IR spectrums of CQD and modified CQD-Fe, the appeared peak at 593 cm^–1^ is related to the stretching vibration, confirming the link of Fe with oxygen contained groups^11^. It seems that the supersaturated solution of Fe^2+^, in the synthesis of the CQD-Fe product, proceeds with nucleation and bonding Fe atoms via carboxylic acid groups to the carbon surface. In other words, Fe atoms which are produced from the disproportionation reaction find the CQD surface as appropriate sites for nucleation and bonding to produce CQD-Fe nanoparticles^9^.

The XRD pattern of the synthesized CQDs is presented in Fig. 4b. The average crystallite size of CQD and CQD-Fe nanoparticle calculated by using Scherrer’s equation according to the relevant XRD peaks were 13.4 and 7.2 nm respectively. Satisfactory agreement between the obtained XRD pattern and a previously reported spectrum is observed^18,27^. Meanwhile, there is no signal for impurities in XRD spectrum for both the nanoparticles.

**Figure 5:**
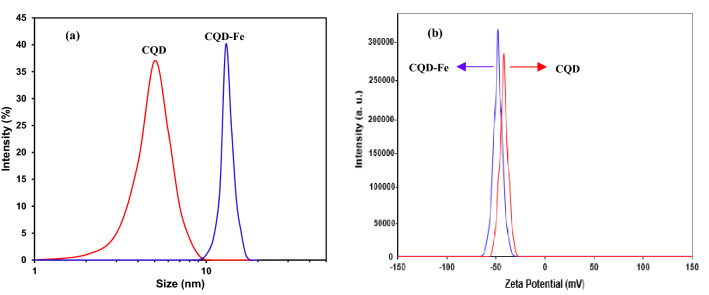
DLS analysis (**a**) and Zeta potential analysis (**b**) for the nanofluids containing 0.001wt% of the CQD and CQD-Fe.

Figure 5a illustrates the particle-size distribution obtained with DLS test. The mean size of the prepared CQD and CDQ-Fe products were appeared about 8.4 and 14.2 nm, respectively. Zeta potential analysis was also employed to measure the surface charge of CQDs. As seen in Fig. 5b, Zeta potential values of –42.4 and –48.3 mV, were corresponding to CQD and CQD-Fe products, respectively.

**Figure 6:**
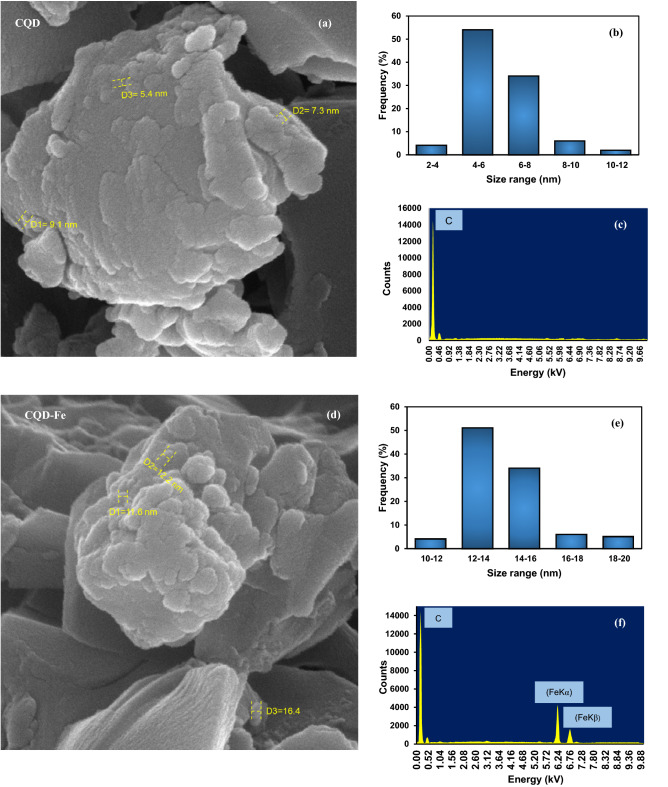
SEM image, particle size distribution and EXD analysis for CQD (**a–c**), and for CQD-Fe (**d–f**).

Figure 6a,d display SEM images of the synthesized CQDs with high uniform and spherical morphology. It can be realized form the depicted results that CQD and CQD-Fe have the average size of 7.9 and 13.8 nm. As presented in particle-size distribution graphs, Fig. 6b,e, more than 94% of CQD nanoparticles were in the range of (3–7) nm and more than 82% of CQD-Fe within the range of (11–14) nm.

In the presented EDX analysis of Fig. 6c,f, iron has two distinguished peaks of K_α_ and K_β_ relevant to electron transitions from the outer shells to the closest shell around the iron nucleus_._ High abundance of carbon, indicated by sharp peaks, confirms non-significant impurity. The unimpressive peak near carbon peak is related to oxygen atoms.

### Hydrodynamic studies

The various drop sizes, generated from different nozzles, are listed in Table 2. Applying pulsed electric fields gives no significant impact on drop size due to the installation of electrodes above the nozzles’ tip. No sensible change was also observed in the terminal velocities by applying electric fields. Adding to this, the size of drops did not change considerably in the presence of different concentrations of either of the CQDs.

**Table 2:** The range of generated drop size using different nozzles.

Nozzle number	Drop size range (mm)					
	1	2	3	4	5	6
CQD nanofluid	2.24–2.66	2.40–2.84	3.06–3.22	3.19–3.45	3.31–3.73	3.42–3.81
CQD-Fe nanofluid	2.19–2.57	2.44–2.82	2.68–3.08	2.87–3.26	3.05–3.43	3.35–3.51

**Figure 7:**
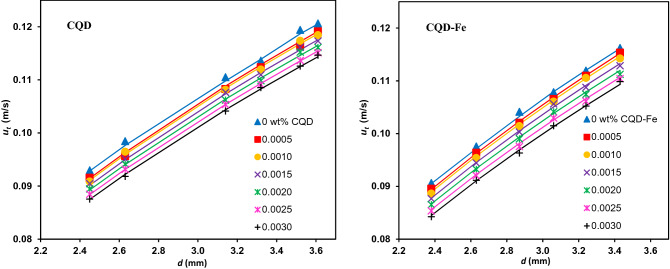
Comparison of the terminal velocity data with the Grace model (continuous lines) for different concentration of CQDs.

Drops are categorizing into three substantial groups of rigid, circulating and oscillating, based on their internal flow pattern. In this regard, circulating state can be identified using the well-known criteria of the dimensionless group H, introduced by Grace et al.^28^, drop Webber number, ${\text{We}} = {{du_{t}^{2} \rho_{c} } \mathord{\left/ {\vphantom {{du_{t}^{2} \rho_{c} } \gamma }} \right. \kern-\nulldelimiterspace} \gamma }$, drop Reynolds number, ${\text{Re}}_{c} = {{\rho_{c} u_{t} d} \mathord{\left/ {\vphantom {{\rho_{c} u_{t} d} {\mu_{c} }}} \right. \kern-\nulldelimiterspace} {\mu_{c} }}$^29^, ratio of ${{{\text{Re}}_{c} } \mathord{\left/ {\vphantom {{{\text{Re}}_{c} } {{\text{N}}_{PG}^{{{ - 0}{\text{.15}}}} }}} \right. \kern-\nulldelimiterspace} {{\text{N}}_{PG}^{{{ - 0}{\text{.15}}}} }}$^30^ ($u_{t}$ is the drop terminal velocity, ${\text{N}}_{PG}$ is the inverse of Morton number), and critical drop size ($d_{cr}$), as the threshold of oscillating flow pattern^31^. Comparing the relevant values with the mentioned criteria range in Table 3 reveals that all drops were in circulating state. Figure 7 demonstrates the increasing trend of terminal velocity as a function of drop size and that there is an excellent agreement of the measured velocities with those predicted with the Grace model.

**Table 3:** The circulating state criteria of drops along with corresponded values^32^.

Criterion	Nanofluid	
	CQD	CQD-Fe
${2}{\kern 1pt} {\kern 1pt} { < }{\kern 1pt} {\kern 1pt} {\text{H}}{\kern 1pt} {\kern 1pt} { < }{\kern 1pt} {\kern 1pt} {59}{\text{.3}}$	12.12–28.04	11.23–25.28
${\text{We}}{\kern 1pt} \;{ < }\;{3}{\text{.58}}$	0.40–1.09	0.35–0.96
${{\text{Re}}}_{c} / {{\text{(N}}}_{PG}^{- 0.15} < 20$	5.28–10.62	4.87–9.75
200 < $\;{\text{Re}}_{c}$ < 500	215.89–428.41	205.45–394.06
$d{\kern 1pt} {\kern 1pt} { < }\,{\kern 1pt} {\kern 1pt} d_{cr}$ (mm)	(2.42–3.61) < 6.47	(2.38–3.43) < 6.19

### Mass transfer studies

The mass transfer performance of the employed system under different conditions was studied through calculating the overall mass transfer coefficient ($K_{od}$) which can be obtained by integrating of a differential mass balance around drops over the contact time (*t*) from the initial to final concentration^1^,

3 $$K_{od} = \frac{ - d}{{6t}}\ln (1 - E)$$

in which, *E* stands for extraction efficiency defined as

4 $$E = \frac{{C_{di} - C_{df} }}{{C_{di} - C_{d}^{*} }}$$

where initial and final solute (acetic acid) concentrations are denoted by $C_{di}$ and $C_{df}$ and $C_{d}^{*}$ denotes the equilibrium solute concentrations in drops with the continuous phase concentration. Considering the much higher continuous phase content in the column (≈ 4 L), compared to the moving single drops (less than 0.01 mL each), the purity of continuous phase was not altered in each series of experiments. Therefore, a negligible $C_{d}^{*}$ value was corresponding.

The determined mass transfer coefficients with CQD and CQD-Fe nanofluids were found within the ranges of (158.4–386.7) and (137.5–325.4) μm/s, having the average enhancements of, respectively, 97.4 and 123.5%, compared to the base fluid with no CQD.

**Figure 8:**
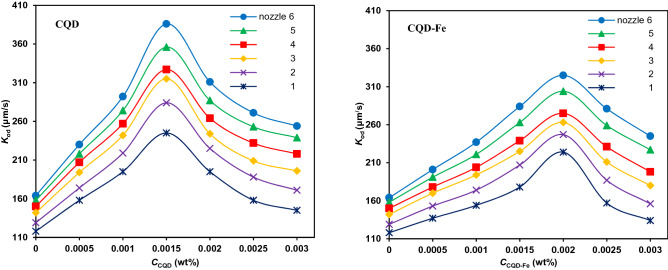
Mass transfer coefficient versus concentration of the CQDs for different drops generated from nozzles with no electric field.

The impact of nanofluids on the overall mass transfer of various drop sizes is illustrated in Fig. 8. Increasing concentration of nanofluids initially improves and then declines the mass transfer coefficient. The enhancing effect of nanofluids in extraction process is based on the random movement of nanoparticles, well known as Brownian motion. However, their movement at high concentrations would be restricted due to the possible aggregation and clustering phenomena. The ongoing fluctuate of nanoparticles disturbances the microconvection inside drops. Another reason is attributed to the excess interfacial resistance due to the obstruction by nanoparticles which made the solute transfer difficult^33^. As it is also obvious, the optimum concentration depends on the size. In this regard, the maximum improvement of mass transfer coefficient for the CQD with the smaller average particle size of 7.2 nm, has been relevant to 0.0015 wt% of the CQD, whereas it is 0.002 wt% for CQD-Fe with the larger average size of 13.4 nm. The possible reason is that there is more aggregation possibility for smaller nanoparticles even at low concentrations.

Based on the molecular dynamic simulation, the influencing factor on Brownian motion, as major mechanism of turbulence and promoted mass transfer, has been quantified with the following equation^34^,

5 $$v = \sqrt {\frac{{3k_{b} T}}{{m_{p} }}} = \sqrt {\frac{{18k_{b} T}}{{\pi \rho_{p} d_{p}^{3} }}}$$

where, $v$, $k_{b}$, $T$, $m_{p}$ and $\rho_{p}$ indicate the velocity of a nanoparticle, Boltzmann constant, temperature, mass and diameter of the particles, respectively. In this regard, at a certain temperature, nanoparticles with smaller size and lower density would result higher mass transfer coefficients. Table 4 presents a comparative list of liquid–liquid extraction systems; precisely CQD-based nanofluids exhibit amazing capability for mass transfer intensification (1.36–8.80 times), which can be attributed to the extremely low density and small size they have. Results also show that just the CQD nanoparticle with maximum mass transfer enhancement of 134.1% has greater potential compared to CQD-Fe due to the desired density and particle size.

**Table 4:** Comparison of the mass transfer enhancements by using different nanofluids.

Extraction column	Nanofluid	Nanoparticle size (nm)	Nanoparticle density (kg/m)	Max. mass transfer enhancement (%)	Ref
Single drop	TiO_2_-water	30–50	4260	40	^35^
	CNT-water	30–50	2216	50	
Pulsed plate	SiO_2_-kerosene	5–30	2150	60	^36^
Rotary disc	SiO_2_-kerosene	3–50	2150	45	^37^
Randomly packed	SiO_2_-toluene	10–80	2150	27–42	^4^
Spray	SiO_2_-toluene	14	2150	47	^38^
Single drop	CQD-kerosene	7.2	1291.4	134.1	This work
Single drop	CQD-Fe-kerosene	13.4	1536.7	105.7	This work

As presented in Fig. 8, the mass transfer coefficient increases with drop size due to tendency of drops to higher internal circulation. Further, the terminal velocity would be higher for large drops and thus, lower contact times will give higher overall mass transfer coefficients.

**Figure 9:**
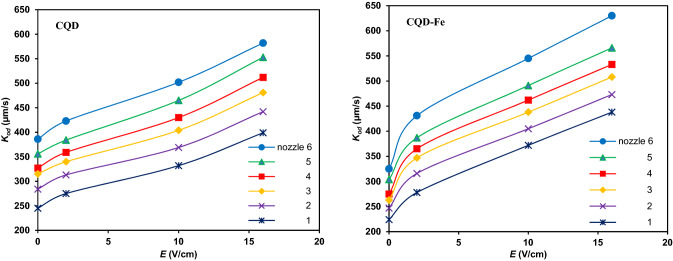
Mass transfer coefficient versus electric field strength under frequencies of 150 (CQD) and 250 Hz (CQD-Fe) for different nozzles and concentrations of 0.0015 (CQD) and 0.002 wt% (CQD-Fe).

The next round of experiments was conducted to investigating the influence of pulsed electric fields on the mass transfer of CQD-based nanofluids. The mass transfer coefficient of CQD and CQD-Fe nanofluids, with optimum concentrations was incredibly promoted to (275.3–582.8) and (278.1–630.3) μm/s, under the pulsed electric field with maximum enhancements of 263.5 and 291.6%, respectively. In this regard, the ascending variation of mass transfer coefficient with pulsed electric field strength, depicted in Fig. 9, indicates that applying external electric field is an efficient approach for boosting the mass transfer performance of CQDs.

Further experiments revealed that the optimum nanoparticle concentration (corresponding to the maximum mass transfer) was desirably shifted to lower values as a consequence of nanoparticle agglomeration when electric fields were used. Thus, the threshold of mass transfer descending appeared at lower nanoparticle concentrations.

As was described above, extraction with conductive nanofluid drops, assisted with pulsed electric field, is relevant to the motion of nanoparticles. In this regard, nanoparticles find electro-induced motions in addition to their inherent random Brownian motion. Thus, displacements of poles in the pulsed electric fields boost the movement of the conductive nanoparticles which in turn induces chaotic advection and highly improves the mass transfer performance^39^.

The effectiveness of pulsed electric fields on mass transfer intensification of nanofluids depends on electrical surface charge of nanoparticles, Zeta potential, and electrical conductivity of the nanofluid. In this regard, the CQDs were modified with bonded iron atoms. The advantage is that applying an electric field will be more effective for the CQD-Fe product with higher Zeta potential of − 48.3 mV compared to − 42.4 mV for the CQD. Use of conductive nanoparticles causes remarkable improvement in the conductivity of nanofluids^40^. Therefore, the CQD-Fe with unique physicochemical properties is preferential.

**Figure 10:**
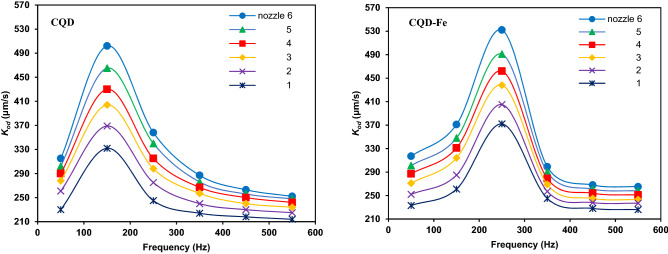
Mass transfer coefficient versus electric field frequency for different drops at typical electric field strength of 10 V/cm and concentrations of 0.0015 (CQD) and 0.002 wt% (CQD-Fe).

Figure 10 shows the effect of electric field frequency on the mass transfer of nanofluids. Results show that by increasing frequency up to an optimum value, mass transfer is improved due to the action of electro-induced motions in which higher frequencies would give subsequent internal microconvection. After an optimum frequency, mass transfer performance will diminish which can be attributed to avoiding efficient movement of nanoparticles at extremely high frequencies in short periods and the fast switching between poles. Thus, there was an optimum point of about 150 Hz and 250 Hz for CQD and CQD-Fe nanofluids. Accordingly, the motion of the smaller size CQD is more influenced with frequency.

It has to mention that mass transfer enhancement through simultaneous use of nanofluids and pulsed electric fields is coincident with economical approach since the power consumption at the highest applied voltage of 80 V (electric field strength of 16 V/cm) was only 0.16 W. The confined continuous phase volume between the electrodes was 0.75 L and thus, the relevant dissipated power per volume was 0.21 W/L. Low voltages also imply safe operations for the operators.

### Comparative study

**Figure 11:**
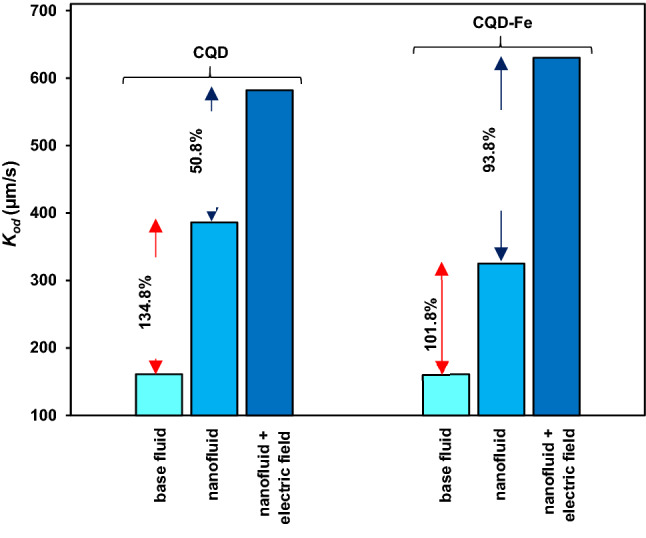
Comparison of mass transfer enhancements of nanofluid drops, with and without pulsed electric field for the largest nozzle No. 6; electric field strength of 16 V/cm and frequencies of 150 and 250 Hz for CQD and CQD-Fe nanofluids.

Comparison between different mass transfer cases including the only base fluid, nanofluids, and applying electric field is presented in Fig. 11 for the largest nozzle No. 6. Interestingly, although CQD-Fe nanofluid exhibits less intensification effect in the absence of pulsed electric field, due to the relatively large particle size and high density, higher mass transfer enhancement was obtained when the pulsed electric field was applied.

**Figure 12:**
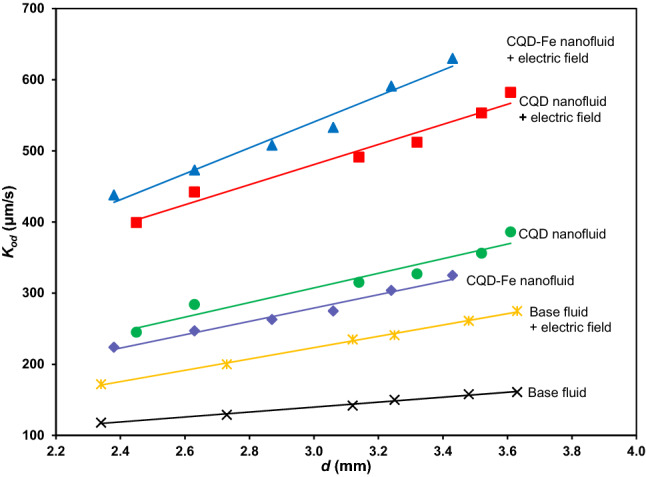
Comparison between different approaches for mass transfer intensification.

In a different presentation, the obtained results versus drop size are presented in Fig. 12. As expected, relatively low mass transfer coefficient was relevant when internal turbulence with drop size was the only effective parameter for the base fluid drops, giving $K_{od}$ values within (118.4–161.7) μm/s. It is while, applying pulsed electric field gives rise the non-uniform distribution of interface accumulated charges and subsequent interfacial instability of drops^26^, leading to $K_{od}$ values of (172.3–275.1) μm/s. As was pointed, utilizing nanofluids with the responsible mechanism of the Brownian motion is likely. In this regard, CQD with smaller particle size and lower density is more efficient than CQD-Fe ($K_{od}$ values of 245.5–386.7 compared to 224.6–325.4 μm/s, respectively). An optimum nanoparticle concentration was appropriate as was described earlier. In the last approach, amazing high $K_{od}$ values of (399.2–582.8) μm/s for CQD and (438.7–630.3) μm/s for CQD-Fe were achieved with simultaneous use of nanofluids and pulsed electric field. By applying the pulsed electric field, the enhancing effect of CQD-Fe were higher than CQD nanofluid which is due to coupling the conductive carbon material with iron atoms having higher Zeta potential and electrical conductivity.

The advances of pulsed electric fields for improving mass transfer coefficient were also evaluated by comparing with those previously reported with different kinds of external field. As tabulated in Table 5, ultrasound and magnetic field external fields could not promote the mass transfer as efficient as pulsed electric field under rather similar conditions (nanofluid circulating drops). This is due to limited impact on global motion of nanoparticles. The capability of pulsed electric fields is confirmed by comparing the mass transfer enhancement results exposed to different external fields. Indeed, utilizing the pulsed electric field with constantly displacement of positive and negative poles brings about higher nanoparticle motions and subsequent internal turbulence of drops. Interestingly, the efficiency of pulsed electric field could be improved by modifying conductive nanoparticles; whereas, the impact of ultrasonic field does not alter with the chemical structure of nanoparticles. Moreover, despite restricting performance of magnetic field to magnetic nature of particles, a variety of conductive nanoparticles could be utilized in the presence of pulsed electric field. Moreover, applying electric filed is coincident with a lower energy consumption and low operating costs.

**Table 5:** Comparison of the impact of different external fields on the mass transfer intensification of circulating drops.

External field characteristics	Nanofluid	Max. mass transfer enhancement (%)	Ref
Magnetic field, 0.36–1.45 T, 41 Hz	Fe_3_O_4_—toluene	121	^11^
Magnetic field, 0.01–0.04 T, 0–500 Hz	Fe_3_O_4_—toluene	259	^12^
Ultrasonic field, 0.37 mW/cm^2^, 35–40 kHz	Fe_3_O_4_—water	79	^10^
Pulsed electric field, 2–16 V/cm, 50–550 Hz	CQD—kerosene	263.5	This work
Pulsed electric field, 2–16 V/cm, 50–550 Hz	CQD-Fe—kerosene	291.6	This work

### Modelling of results

The influence of pulsed electric field, size and surface charge and concentration of nanoparticles, physical properties of the chemical system, and drop size can be involved in a wide-ranging and accurate model for predicting the mass transfer enhancements. Meanwhile, low slope of the equilibrium curve for the used chemical system at 20 °C, ($m \approx$ 0.09) within the acetic acid concentration range confirms that the mass transfer resistance lies in the dispersed phase^41^ i.e. $K_{od} \approx k_{d}$ according to the Whitman two film theory.

Among the several mass transfer models, proposed for various hydrodynamic conditions, the stagnant spherical drops with dominant molecular diffusion and the circulating drops with the assumption of laminar internal circulation have been frequently employed by using the Newman^42^ and the Kronig and Brink^43^ models, respectively. However, both the models are not suitable for drops in this study due to the circulating mode and relatively high Reynolds number. Here, the comprehensive Kumar and Hartland correlation with nice ability of predicting the Sherwood number of dispersed phase of circulating drops in different chemical systems, ${\text{Sh}}_{d}$, can be utilized^44^:

6 $${\text{Sh}}_{d} = \frac{{k_{d} d}}{{D_{d} }} = 17.7 + \alpha \frac{{3.19 \times 10^{ - 3} ({\text{Re}}_{c} {\text{Sc}}_{d}^{{1/3}} )^{1.7} }}{{1 + 1.43 \times 10^{ - 2} ({\text{Re}}_{c} {\text{Sc}}_{d}^{{1/3}} )^{0.7} }}\left( {\frac{{\rho_{d} }}{{\rho_{c} }}} \right)^{2/3} \frac{1}{{1 + \left( {{{\mu_{d} } \mathord{\left/ {\vphantom {{\mu_{d} } {\mu_{c} }}} \right. \kern-\nulldelimiterspace} {\mu_{c} }}} \right)^{{{2 \mathord{\left/ {\vphantom {2 3}} \right. \kern-\nulldelimiterspace} 3}}} }}$$

in which ${\text{Sc}}_{d} = {{\mu_{d} } \mathord{\left/ {\vphantom {{\mu_{d} } {\rho_{d} D_{d} }}} \right. \kern-\nulldelimiterspace} {\rho_{d} D_{d} }}$ denotes dispersed phase Schmidt number and $\alpha$ is defined as the enhancement factor of intensification mass transfer of drops. Predicting mass transfer coefficients for some intensification methods like use of nanoparticles or external fields is viable by this factor, exerted for the second term of the equation to take into account the applied conditions. Here, the enhancement factors for the only base fluid were found close to unity, indicating excellent agreement with the data. The precise ability of this correlation has been demonstrated through satisfactory prediction of mass transfer performance in different extraction set-ups and chemical systems in recent investigations^12,45,46^.

The calculated $\alpha$ values based on the above equation and accord to the relevant parameter values for the simultaneous effects of pulsed electric field and nanofluids were within (1.82–2.84) and (2.10–3.62) for CQD and CQD-Fe nanofluids, respectively. Correlating this enhancement factor, $\alpha$, is available in terms of pertinent dimensionless parameters.

The enhancing effect of pulsed electric field is directly proportional to the surface charge of nanoparticles corresponding to the electrical double layer and Zeta potential. Accordingly, the main variables of the dielectric constant of the base fluid ($\varepsilon_{f}$), the Zeta potential ($\xi$) as the potential alteration between the continuous phase and the nanofluid, nanoparticle radius (*r*) and the electric double layer on particles ($\lambda_{D}$) determine the electric surface charge ($q_{p}$), as

7 $$q_{p} = \varepsilon_{f} \xi \left( {\frac{1}{r} + \frac{1}{{\lambda_{D} }}} \right)\pi r^{2}$$

A dimensionless parameter, $L^{*}$, as the as the ratio of electro-induced motion velocity to an average terminal velocity (${{l_{c} } \mathord{\left/ {\vphantom {{l_{c} } {\overline{t}}}} \right. \kern-\nulldelimiterspace} {\overline{t}}}$) and involving the impact of both the surface charge of nanoparticle and the electric field strength can be introduced as^15^

8 $$L^{*} = \frac{{q_{p} E\overline{t}}}{{6\pi \mu_{f} rl}}$$

in which, the surface charge of nanoparticles and the pulsed electric field strength are demonstrated with $q_{p}$ and $E$, respectively. $\overline{t}$ and $l$ are the average contact time and the passing distance of drops along the column.

Considering the frequency of pulsed electric field, a dimensionless variable with the aid of heat and mass transfer analogy can been provided as

9 $$\omega^{*} = \frac{{\omega \;d^{2} }}{{4D_{d} }}$$

Here, replacing the thermal diffusivity with the analog molecular diffusivity, the inverse of Fourier number in heat transfer is resembled. Abdelaal and Jog have competently investigated the analogy of heat and mass transfer of drops under electric fields^47^.

**Figure 13:**
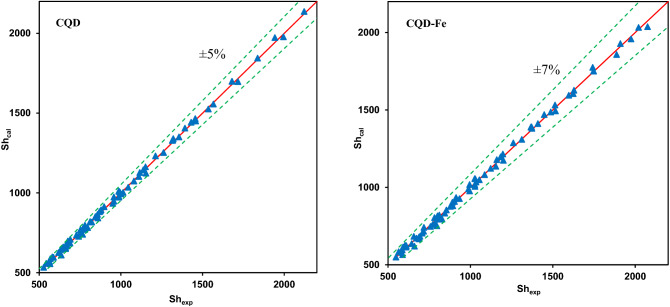
Comparison of the predicted and experimental Sherwood numbers.

The correlation of enhancement factor was expressed in terms of the above mentioned variables as well as $C_{{{\text{CQD}}}}$ (or $C_{{\text{CQD - Fe}}}$) and the drops Reynolds number. Developing the regression analysis of CQD and CQD-Fe nanofluids using Origin 2019 software, the following best fitting correlation were found,

10 $$\alpha { = 1 + 0.431(0.427C_{{{\text{CQD}}}}^{0.64} - 0.003C_{{{\text{CQD}}}}^{0.33} )^{1.94} (0.234\omega^{{*0.67 }} - 0.007\omega^{*0.92} )^{2.03} L^*1.12 {\text{Re}}^{{{0.23}}}}$$

11 $$\alpha { = 1 + 0.345(0.387}C_{{\text{CQD - Fe}}}^{0.53} - 0.004C_{{\text{CQD - Fe}}}^{0.27} )^{1.87} (0.512\omega^{{*0.71 }} - 0.0.009\omega^{*1.04} )^{2.24} L^{{*1.67 }} {\text{Re}}^{0.29}$$

for CQD and CQD-Fe nanofluids, respectively. The acquired close to unity determination coefficient (${\text{R}}^{{2}}$) values of 0.991 and 0.987 in addition to the adjusted coefficient of determination (${\text{R}}_{{{\text{adj}}}}^{{2}}$) of 0.988 and 0.985 for CQD and CQD-Fe nanofluids, respectively, confirm that the experimental data could be reproduced by these fitting models. The symmetrical distribution of experimental data and those predicted (by Eqs. 6, 10 or 11) around the square diagonal (Fig. 13) and the maximum relative deviation of ± 5% and ± 7% for CQD and CQD-Fe nanofluids, respectively; again confirm that the proposed model is satisfactory.

## Conclusion

This study presented the performance of the synthesized carbon quantum dots together with the pulsed electric field in the mass transfer intensification. Two steps were involved namely (i) examining only CQD nanofluids, and (ii) examining simultaneous use of nanofluids and electric fields. In both the cases, results revealed a close agreement of the terminal velocity of drops with theoretical bases indicating no sensible alternation in the hydrodynamic behavior of drops with low CQD doses and low voltage pulsed electric fields. It was while, the mass transfer rate was primary increased with only the nanofluids to more than one-fold enhancements and then decreased at high CQD concentrations. The remarkable improvement, in this case, can be attributed to the extremely small size and low density of CQDs assisting the Brownian motion. Upon applying the low voltage pulsed electric fields on nanofluids, the CQD motions was strongly improved due to the adjustable electro-induced motions toward the switching opposite charge electrode. Accordingly, about three folds mass transfer enhancements were achieved this time. The priority of modified CQD-Fe was obvious due to the higher Zeta potential and electrical conductivity. Thus, the simultaneous use of pulsed electric field and carbon quantum dot nanofluids seems as a novel method for mass transfer intensification. An accurate model for predicting mass transfer coefficient according to the Kumar and Hartland correlation, accompanied with the proposed correlations of enhancement factor and in terms of involved variables, could precisely represent the results.

## Data Availability

Correspondence and requests for data and materials should be addressed to J.S.
